# Supplementary material for: Comparative Analysis of How the Fecal Microbiota of Green-Winged Saltator (Saltator similis) Diverge among Animals Living in Captivity and in Wild Habitats
Source: Animals (Basel). 2024 Mar 19;14(6):937. doi: 10.3390/ani14060937 (PMC10967636; doi:10.3390/ani14060937)
Supplement: Supplementary file 1 [file animals-14-00937-s001.zip › Suplementary Figures.pdf]

List of Supplementary Figures

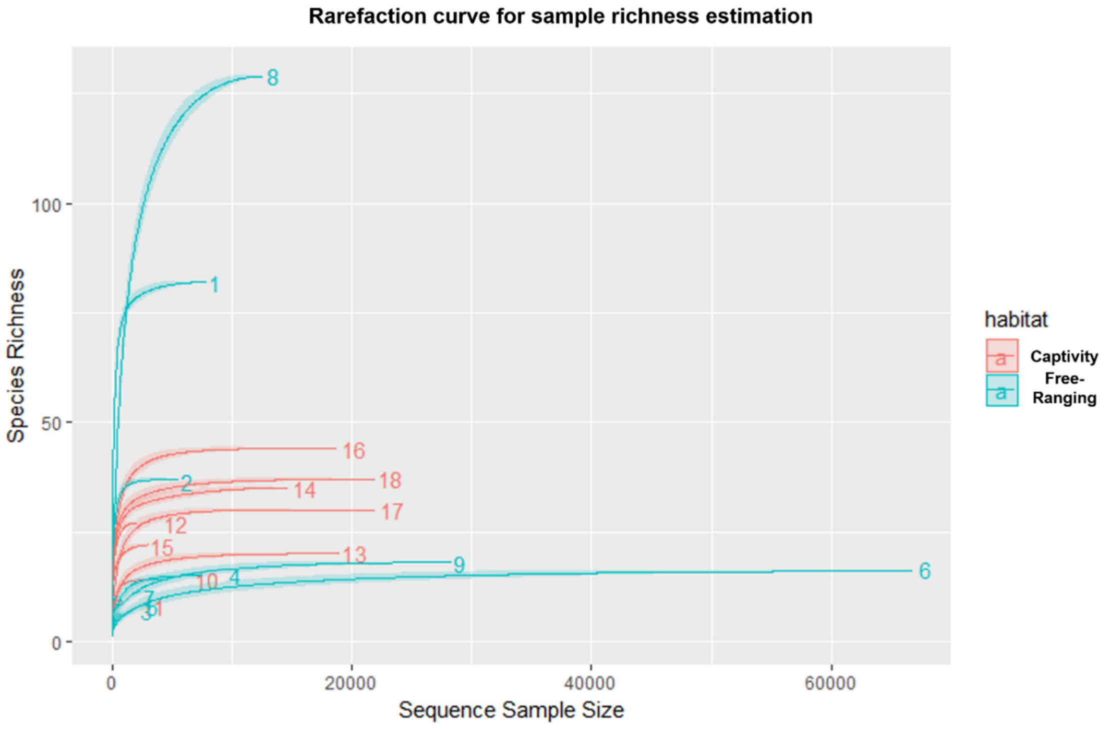

Supplementary Figure S1 The rarefaction curves of the sequences from *Saltator similis*. The lines represent each one of the sequenced libraries. The turquoise lines represent de libraries from free-ranging *S. similis*, and the pink lines represent libraries from captivity *S. similis*.

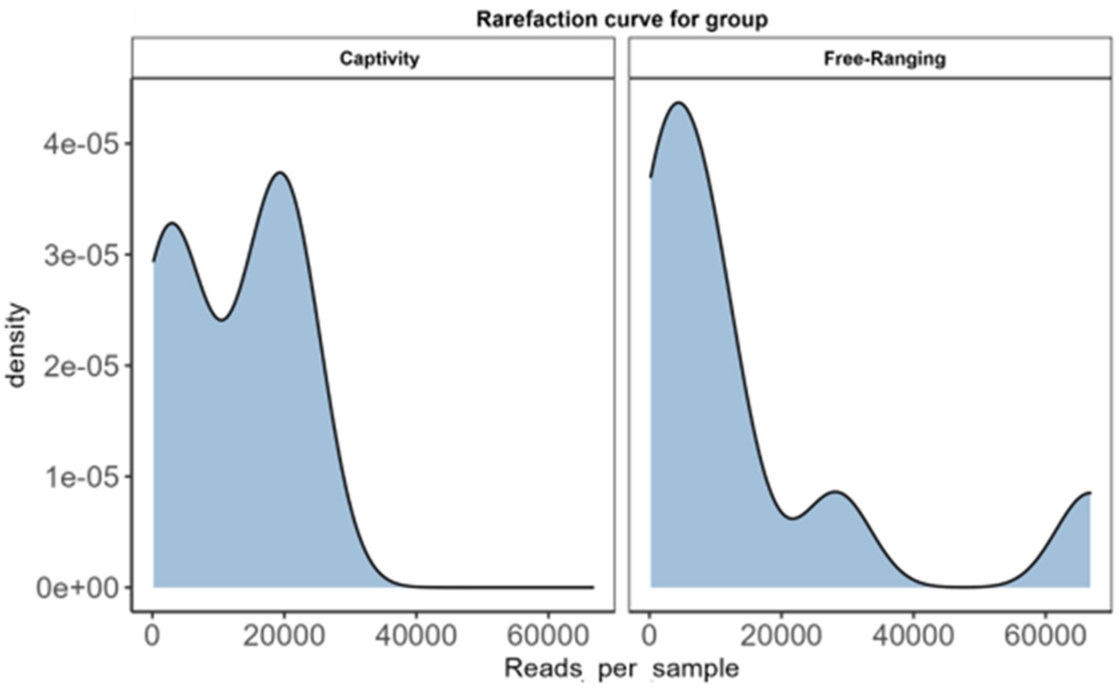

Supplementary Figure S2 The rarefaction curves of the sequences from *Saltator similis*, per group. Comparison of the identified ASVs with the number of sequenced reads in each group.

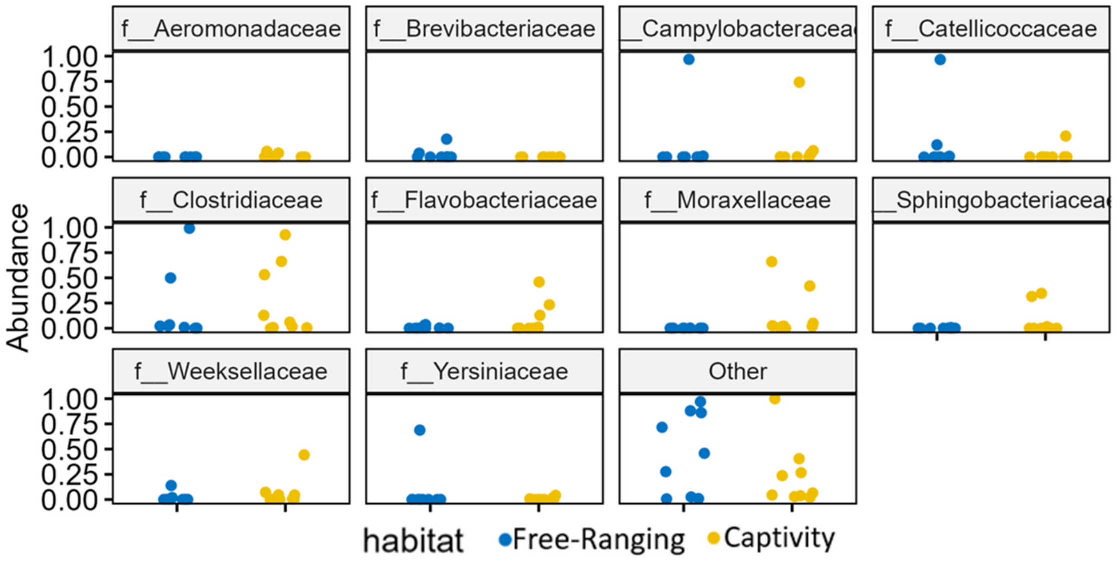

Supplementary Figure S3 Relative abundance of the principal bacterial families identified in the feces of all studied *Saltator similis*. The points represent the ASVs. Blue color: Free-ranging songbirds. Yellow color: captivity songbirds

	Captivity	Free-ranging
**Phyla**	*Firmicutes*<br>*Bacteroidota*<br>*Proteobacterias*<br>*Actnobacteriota*	*Firmicutes*<br>*Bacteroidota*<br>*Proteobacterias*<br>*Actnobacteriota*
**Family**	*Weeksellaceae*<br>*Micrococcaceae*<br>*Lachnospiraceae*<br>*Campylobacteriaceae*<br>*Clostridiaceae*<br>*Catelliococcaceae*<br>*Moraxellaceae*<br>***Sphingobacteriaceae****<br>***Flavobacteriaceae****	*Weeksellaceae*<br>*Micrococcaceae*<br>*Lachnospiraceae*<br>*Campylobacteriaceae*<br>*Clostridiaceae*<br>*Catelliococcaceae*<br>*Moraxellaceae*<br>***Mycoplasmataceae****<br>***Corynebacteriacea****
**Genera**	*Anaerosporobacter*<br>*Campylobacter*<br>***Aeromonas****<br>***Acinetobacter****<br>***Empedobacter****<br>***Flavobacterium****<br>***"Candidatus* Arthromitus"***<br>***Sphingobacterium****<br>***Acidibacter****	*Anaerosporobacter*<br>*Campylobacter*<br>***Catelliococcus****<br>***Actinobacillus****<br>***Brevibacterium****<br>***Clostridium* sensu stricto 1***<br>***Serratia****<br>***Mycoplasma****

Supplementary Figure S4 The most predominant phyla, family, and genera identified in the feces from captivity and free-ranging *Saltator similis*. Bold *: Indicates family or genus identified in libraries from a single habitat. Results from RStudio using packages Vegan and the function Adonis and Adonis2.
